# Supplementary figures and images for: Emotional flexibility and general self-efficacy: A pilot training intervention study with knowledge workers
Source: PLoS One. 2020 Oct 14;15(10):e0237821. doi: 10.1371/journal.pone.0237821 (PMC7556510; doi:10.1371/journal.pone.0237821)

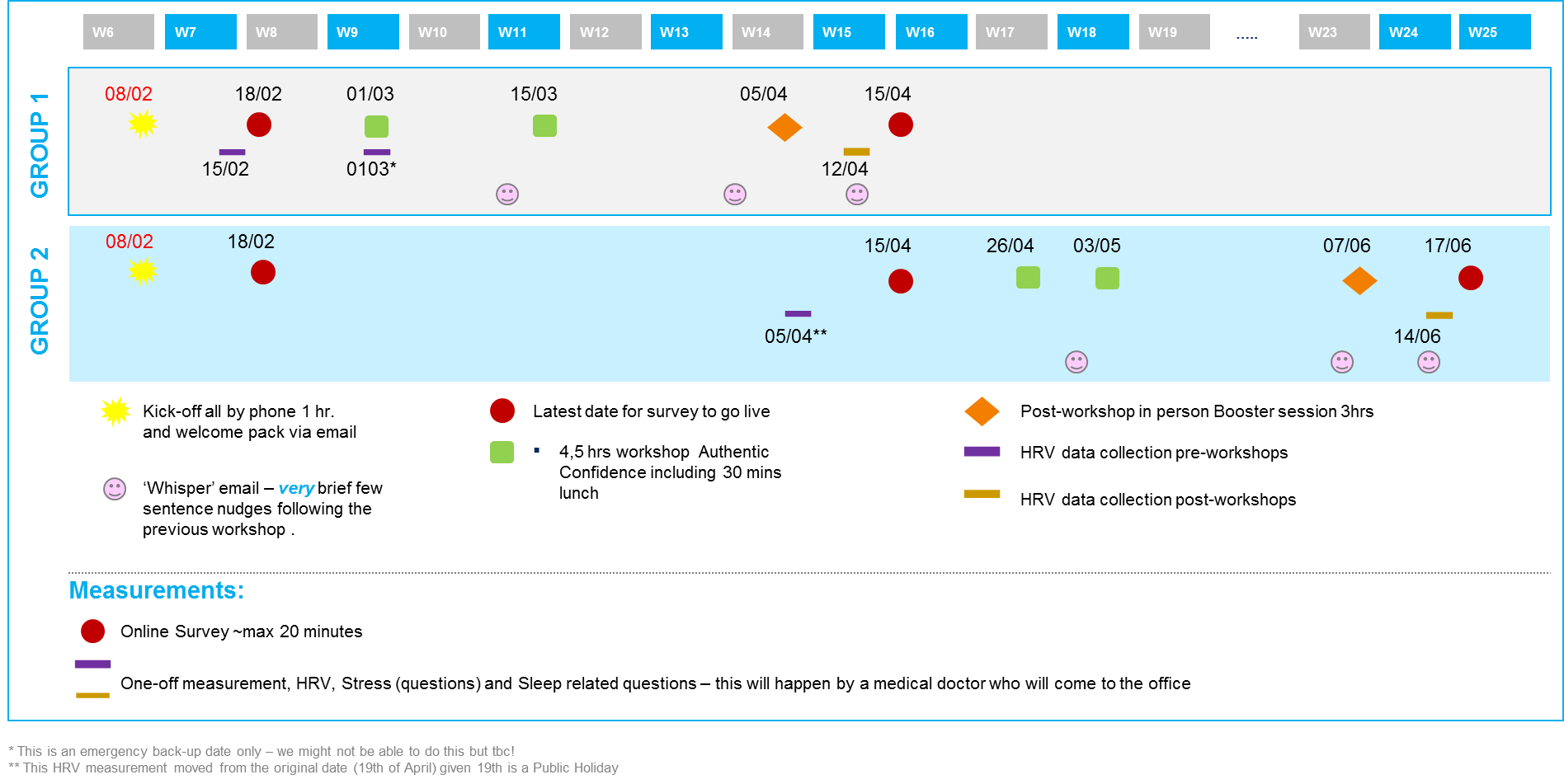

Supplement: S1 Fig — (TIF) [file pone.0237821.s001.tif]

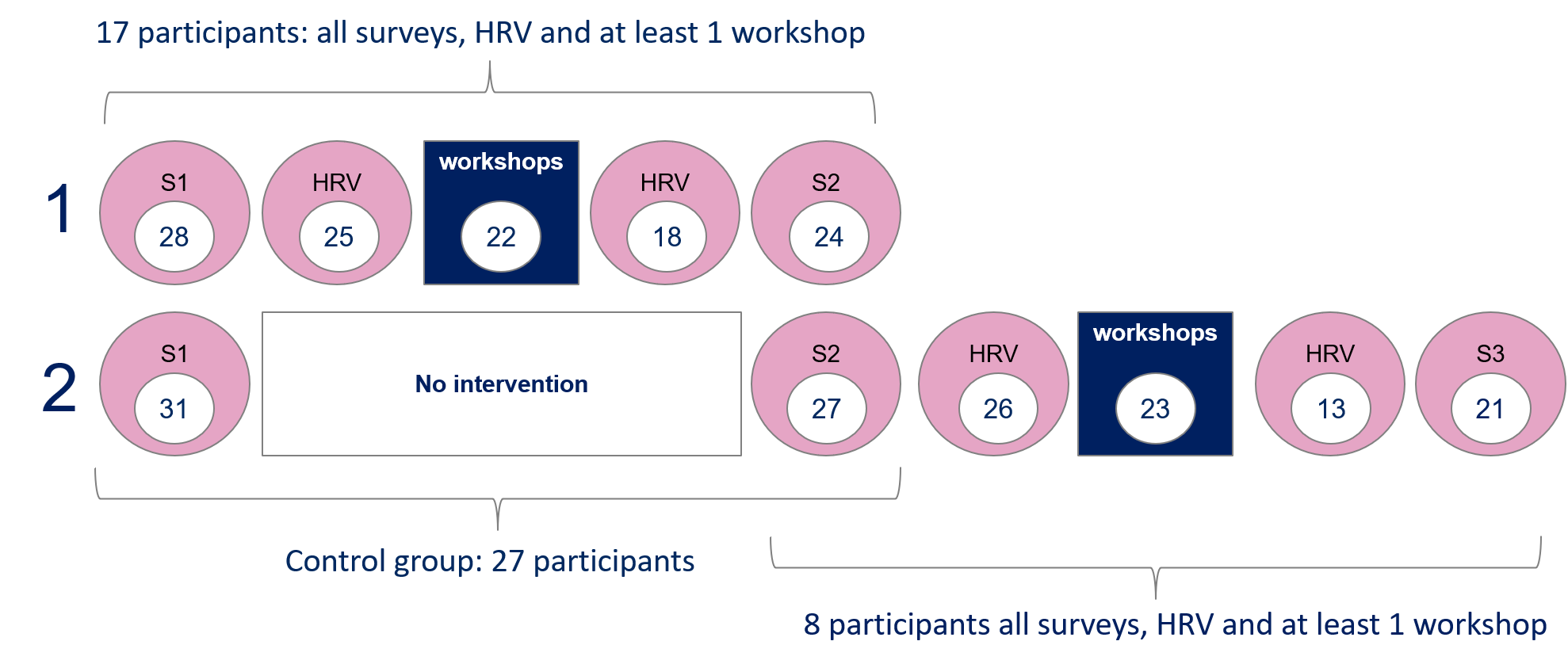

Supplement: S2 Fig — (TIF) [file pone.0237821.s002.tif]
