# Supplementary material for: Emotional flexibility and general self-efficacy: A pilot training intervention study with knowledge workers
Source: PLoS One. 2020 Oct 14;15(10):e0237821. doi: 10.1371/journal.pone.0237821 (PMC7556510; doi:10.1371/journal.pone.0237821)
Supplement: S1 Appendix — (DOCX) [file pone.0237821.s003.docx]

S1 Appendix

**Emotional Flexibility (EF) and Subscales**

| **Abbr** | **Dimension** | **Official Scale** | **Item** |
| --- | --- | --- | --- |
| CA1 | EF | COMMITTED ACTION | CommAction1_1 Even when I stumbled in my efforts, I didn't quit working towards what is important |
| CA2 | EF | COMMITTED ACTION | CommAction1_2 Even when times got tough, I was still able to take steps toward what I value in life |
| CA3 | EF | COMMITTED ACTION | CommAction1_3 Even when life got stressful and hectic, I still worked toward things that were important to me |
| CA4 | EF | COMMITTED ACTION | CommAction1_4 I didn't let setbacks slow me down in taking action toward what I really want in life |
| CA5 | EF | COMMITTED ACTION | CommAction1_5 I didn't let my own fears and doubts get in the way of taking action toward my goals |
| V1 | EF | VALUES | Purpose1_1 I was very in touch with what is important to me and my life |
| V2 | EF | VALUES | Purpose1_2 I tried to connect with what is truly important to me on a daily basis |
| V3 | EF | VALUES | Purpose1_3 I stuck to my deeper priorities in life |
| V4 | EF | VALUES | Purpose1_4 Even when it meant making tough choices, I still tried to prioritize the things that were important to me |
| V5 | EF | VALUES | Purpose1_5 My deeper values consistently gave direction to my life |
| D1 | EF | DEFUSION | Defusion1_1 I was able to let negative feelings come and go without getting caught up in them |
| D2 | EF | DEFUSION | Defusion1_2 When I was upset, I was able to let those negative feelings pass through me without clinging to them |
| D3 | EF | DEFUSION | Defusion1_3 When I was scared or afraid, I was able to gently experience those feelings, allowing them to pass |
| D4 | EF | DEFUSION | Defusion1_4 I was able to step back and notice negative thoughts and feelings without reacting to them |
| D5 | EF | DEFUSION | Defusion1_5 In tough situations, I was able to notice my thoughts and feelings without getting overwhelmed by them |
| SACX1 | EF | SELF AS CONTEXT | Self_as_Context1_1 I tried to keep perspective even when life knocked me down |
| SACX2 | EF | SELF AS CONTEXT | Self_as_Context1_2 When I was scared or afraid, I still tried to see the larger picture |
| SACX3 | EF | SELF AS CONTEXT | Self_as_Context1_3 Even when I felt hurt or upset, I tried to maintain a broader perspective |
| SACX4 | EF | SELF AS CONTEXT | Self_as_Context1_4 When something painful happened, I tried to take a balanced view of the situation |
| SACX5 | EF | SELF AS CONTEXT | Self_as_Context1_5 I carried myself through though moments by seeing my life from a larger viewpoint |
| M1 | EF | PRESENT MOMENT AWARENESS | Mindfulness1_1 I paid close attention to what I was thinking and feeling |
| M2 | EF | PRESENT MOMENT AWARENESS | Mindfulness1_2 I was attentive and aware of my emotions |
| M3 | EF | PRESENT MOMENT AWARENESS | Mindfulness1_3 I was in touch with the ebb and flow of my thoughts and feelings |
| M4 | EF | PRESENT MOMENT AWARENESS | Mindfulness1_4 I was in tune with my thoughts and feelings from moment to moment |
| M5 | EF | PRESENT MOMENT AWARENESS | Mindfulness1_5 I strived to remain mindful and aware of my own thoughts and emotions |
| A1 | EF | ACCEPTANCE | Acceptance1_1 I made room to fully experience negative thoughts and emotions, breathing them in rather than pushing them away |
| A2 | EF | ACCEPTANCE | Acceptance1_2 When I had an upsetting thought or emotion, I tried to give it space rather than ignoring it |
| A3 | EF | ACCEPTANCE | Acceptance1_3 I was receptive to observing unpleasant thoughts and feelings without interfering with them |
| A4 | EF | ACCEPTANCE | Acceptance1_4 I tried to make peace with my negative thoughts and feelings rather than resisting them |
| A5 | EF | ACCEPTANCE | Acceptance1_5 I opened myself to all off my feelings, the good and the bad |

**Emotional Inflexibility (EI) and Subscales**

| **Abbr** | **Dimension** | **Official Scale** | **Item** |
| --- | --- | --- | --- |
| F1 | EI | FUSION | Fusion1_1 Negative thoughts and feelings tended to stick with me for a long time |
| F2 | EI | FUSION | Fusion1_2 Distressing thoughts tended to spin around in my mind like a broken record |
| F3 | EI | FUSION | Fusion1_3 It was very easy to get trapped into unwanted thoughts and feelings |
| F4 | EI | FUSION | Fusion1_4 When I had negative thoughts or feelings, it was very hard to see past them |
| F5 | EI | FUSION | Fusion1_5 When something bad happened, it was hard for me to stop thinking about it |
| LM1 | EI | LACK OF CONTACT WITH VALUES | NotMindful1_1 I did most things on 'automatic' with little awareness of what I was doing |
| LM2 | EI | LACK OF CONTACT WITH VALUES | NotMindful1_2 I went through most days on autopilot without paying much attention to what I was thinking or feeling |
| LM3 | EI | LACK OF CONTACT WITH VALUES | NotMindful1_3 I floated through most days without paying much attention |
| LM4 | EI | LACK OF CONTACT WITH VALUES | NotMindful1_4 I did most things mindlessly without paying much attention |
| LM5 | EI | LACK OF CONTACT WITH VALUES | NotMindful1_5 Most of the time, I was just going through the motions without paying much attention |
| LV1 | EI | LACK OF CONTACT WITH THE PRESENT MOMENT | NoPurpose1_1 My priorities and values often fell by the wayside in my day-to-day life |
| LV2 | EI | LACK OF CONTACT WITH THE PRESENT MOMENT | NoPurpose1_2 The things that I value the most often fell off my prioritiy list completely |
| LV3 | EI | LACK OF CONTACT WITH THE PRESENT MOMENT | NoPurpose1_3 When life got hectic, I often lost touch with the things I value |
| LV4 | EI | LACK OF CONTACT WITH THE PRESENT MOMENT | NoPurpose1_4 I didn't usually have time to focus on the things that are really important to me |
| LV5 | EI | LACK OF CONTACT WITH THE PRESENT MOMENT | NoPurpose1_5 When times got tough, it was easy to forget about what I truly value |
| I1 | EI | INACTION | Inaction1_1 Negative feelings often trapped me in inaction |
| I2 | EI | INACTION | Inaction1_2 Getting upset left me stuck and inactive |
| I3 | EI | INACTION | Inaction1_3 Negative feelings easily stalled out my plans |
| I4 | EI | INACTION | Inaction1_4 Negative experiences derailed me from what's really important |
| I5 | EI | INACTION | Inaction1_5 Unpleasant thoughts and feelings easily overwhelmed my efforts to deepen my life |
| EA1 | EI | EXPERIENTIAL AVOIDANCE | Avoidance1_1 When I had a bad memory, I tried to distract myself to make it go away |
| EA2 | EI | EXPERIENTIAL AVOIDANCE | Avoidance1_2 When unpleasant memories came to me, I tried to put them out of my mind |
| EA3 | EI | EXPERIENTIAL AVOIDANCE | Avoidance1_3 I tried to distract myself when I felt unpleasant emotions |
| EA4 | EI | EXPERIENTIAL AVOIDANCE | Avoidance1_4 When something upsetting came up, I tried very hard to stop thinking about it |
| EA5 | EI | EXPERIENTIAL AVOIDANCE | Avoidance1_5 If there was something I didn't want to think about, I would try many things to get it out of my mind |
| SACN1 | EI | SELF AS CONTENT | Self_as_Content1_1 I thought some of my emotions were bad or inappropriate and I shouldn't feel them |
| SACN2 | EI | SELF AS CONTENT | Self_as_Content1_2 I believed some of my thoughts are abnormal or bad and I shouldn't think that way |
| SACN3 | EI | SELF AS CONTENT | Self_as_Content1_3 I told myself that I shouldn't be feeling the way I'm feeling |
| SACN4 | EI | SELF AS CONTENT | Self_as_Content1_4 I told myself I shouldn't be thinking the way I was thinking |
| SACN5 | EI | SELF AS CONTENT | Self_as_Content_5 I criticized myself for having irrational or inappropriate emotions |

**General Self-Efficacy (GSE)**

| **Abbr** | **Dimension** | **Official Scale** | **Item** |
| --- | --- | --- | --- |
| GSE | GSE | General Self-Efficacy | I can always manage to solve difficult problems if I try hard enough |
| GSE | GSE | General Self-Efficacy | If someone opposes me, I can find the means and ways to get what I want |
| GSE | GSE | General Self-Efficacy | It is easy for me to stick to my aims and accomplish my goals |
| GSE | GSE | General Self-Efficacy | I am confident that I could deal efficiently with unexpected events |
| GSE | GSE | General Self-Efficacy | Thanks to my resourcefulness, I know how to handle unforeseen situations |
| GSE | GSE | General Self-Efficacy | I can solve most problems if I invest the necessary effort |
| GSE | GSE | General Self-Efficacy | I can remain calm when facing difficulties because I can rely on my coping abilities |
| GSE | GSE | General Self-Efficacy | When I am confronted with a problem, I can usually find several solutions |
| GSE | GSE | General Self-Efficacy | If I am in trouble, I can usually think of a solution |
| GSE | GSE | General Self-Efficacy | I can usually handle whatever comes my way |
