# Supplementary material for: Emotional flexibility and general self-efficacy: A pilot training intervention study with knowledge workers
Source: PLoS One. 2020 Oct 14;15(10):e0237821. doi: 10.1371/journal.pone.0237821 (PMC7556510; doi:10.1371/journal.pone.0237821)
Supplement: S2 Appendix — (DOCX) [file pone.0237821.s004.docx]

S2 Appendix

|  | Component |  |  |  |  |  |  |  |  |  |  |
| --- | --- | --- | --- | --- | --- | --- | --- | --- | --- | --- | --- |
|  | 1 | 2 | 3 | 4 | 5 | 6 | 7 | 8 | 9 | 10 | 11 |
| CA2 | 0.748 |  |  |  |  |  |  |  |  |  |  |
| V4 | 0.727 |  |  |  |  |  |  |  |  |  |  |
| CA3 | 0.687 |  |  |  |  |  |  |  |  |  |  |
| V3 | 0.632 |  |  |  |  |  |  |  |  |  |  |
| V2 | 0.632 |  |  |  |  |  |  |  |  |  |  |
| CA4 | 0.622 |  |  |  |  |  |  |  |  |  |  |
| V5 | 0.533 |  |  |  |  |  |  |  |  |  |  |
| V1 | 0.525 |  |  |  |  |  |  |  |  |  |  |
| CA1 | 0.519 |  |  |  |  |  |  |  |  |  |  |
| CA5 | 0.513 |  |  |  |  |  |  |  |  |  |  |
| SACN4 |  | 0.841 |  |  |  |  |  |  |  |  |  |
| SACN3 |  | 0.828 |  |  |  |  |  |  |  |  |  |
| SACN1 |  | 0.809 |  |  |  |  |  |  |  |  |  |
| SACN2 |  | 0.724 |  |  |  |  |  |  |  |  |  |
| SACN5 |  | 0.713 |  |  |  |  |  |  |  |  |  |
| LM3 |  |  | 0.890 |  |  |  |  |  |  |  |  |
| LM4 |  |  | 0.865 |  |  |  |  |  |  |  |  |
| LM2 |  |  | 0.849 |  |  |  |  |  |  |  |  |
| LM1 |  |  | 0.811 |  |  |  |  |  |  |  |  |
| LM5 |  |  | 0.809 |  |  |  |  |  |  |  |  |
| A2 |  |  |  | 0.910 |  |  |  |  |  |  |  |
| A1 |  |  |  | 0.858 |  |  |  |  |  |  |  |
| A3 |  |  |  | 0.711 |  |  |  |  |  |  |  |
| A4 |  |  |  | 0.663 |  |  |  |  |  |  |  |
| A5 |  |  |  | 0.515 |  |  |  |  |  | 0.382 |  |
| A4 |  |  |  | 0.465 |  |  |  | -0.388 |  |  |  |
| LV5 |  |  |  |  | 0.912 |  |  |  |  |  |  |
| LV3 |  |  |  |  | 0.891 |  |  |  |  |  |  |
| LV4 |  |  |  |  | 0.872 |  |  |  |  |  |  |
| LV1 |  |  |  |  | 0.813 |  |  |  |  |  |  |
| LV2 |  |  |  |  | 0.801 |  |  |  |  |  |  |
| I2 |  |  |  |  |  | 0.940 |  |  |  |  |  |
| I1 |  |  |  |  |  | 0.939 |  |  |  |  |  |
| I3 |  |  |  |  |  | 0.912 |  |  |  |  |  |
| I4 |  |  |  |  |  | 0.786 |  |  |  |  |  |
| I5 |  |  |  |  |  | 0.778 |  |  |  |  |  |
| EA1 |  |  |  |  |  |  | 0.892 |  |  |  |  |
| EA3 |  |  |  |  |  |  | 0.846 |  |  |  |  |
| EA2 |  |  |  |  |  |  | 0.820 |  |  |  |  |
| EA5 |  |  |  |  |  |  | 0.713 |  |  |  | 0.378 |
| EA4 |  |  |  |  |  |  | 0.673 |  |  |  | 0.388 |
| F4 |  |  |  |  |  |  |  | 0.734 |  |  |  |
| F3 |  | 0.343 |  |  |  |  |  | 0.690 |  |  |  |
| F2 |  |  |  |  |  |  |  | 0.652 |  |  |  |
| F5 |  |  |  |  |  |  |  | 0.640 |  |  |  |
| F1 |  |  |  |  |  |  |  | 0.561 |  | 0.327 |  |
| D5 |  |  |  | 0.369 |  |  |  | -0.440 |  |  | -0.356 |
| D3 |  |  |  |  |  |  |  | -0.428 |  | 0.366 |  |
| D1 |  |  |  |  |  |  |  | -0.395 |  |  | -0.377 |
| SACX2 |  |  |  |  |  |  |  |  | -0.829 |  |  |
| SACX1 |  |  |  |  |  |  |  |  | -0.803 |  |  |
| SACX3 |  |  |  |  |  |  |  |  | -0.700 |  |  |
| SACX4 |  |  |  |  |  |  |  |  | -0.628 |  |  |
| SACX5 |  |  |  |  |  |  |  |  | -0.619 |  |  |
| D2 |  |  |  |  |  |  |  |  | -0.347 | 0.316 |  |
| M5 |  |  |  |  |  |  |  |  |  | 0.738 |  |
| M2 |  |  |  |  |  |  |  |  |  | 0.713 |  |
| M3 |  |  |  |  |  |  |  |  |  | 0.688 |  |
| M1 |  |  |  |  |  |  |  |  |  | 0.666 |  |
| M4 |  |  |  | 0.395 |  |  |  |  |  | 0.463 |  |

"Extraction Method: Principal Component Analysis. Rotation Method: Oblimin with Kaiser Normalization."

a. Rotation converged in 24 iterations.
