# Supplementary material for: Emotional flexibility and general self-efficacy: A pilot training intervention study with knowledge workers
Source: PLoS One. 2020 Oct 14;15(10):e0237821. doi: 10.1371/journal.pone.0237821 (PMC7556510; doi:10.1371/journal.pone.0237821)
Supplement: S3 Appendix — (DOCX) [file pone.0237821.s005.docx]

S3 Appendix

|  | Component |  |  |  |  |  |  |  |  |  |  |  |
| --- | --- | --- | --- | --- | --- | --- | --- | --- | --- | --- | --- | --- |
| Q | 1 | 2 | 3 | 4 | 5 | 6 | 7 | 8 | 9 | 10 | 11 | 12 |
| CA2 | 0.753 |  |  |  |  |  |  |  |  |  |  |  |
| CA4 | 0.709 |  |  |  |  |  |  |  |  |  |  |  |
| CA5 | 0.618 |  |  |  |  |  |  |  |  |  |  |  |
| CA1 | 0.576 |  |  |  |  |  |  |  |  |  |  |  |
| CA3 | 0.550 |  |  |  |  |  |  |  |  |  |  |  |
| V2 | 0.497 |  |  |  |  |  |  |  |  |  |  |  |
| V3 | 0.470 |  |  |  |  |  |  |  |  |  |  |  |
| V4 | 0.463 |  |  |  |  |  |  |  |  | 0.420 |  |  |
| V1 | 0.368 |  |  |  |  |  |  |  |  |  |  |  |
| V5 | 0.345 |  |  |  |  |  |  |  |  | 0.330 |  |  |
| F4 |  | 0.816 |  |  |  |  |  |  |  |  |  |  |
| F3 |  | 0.745 |  |  |  |  |  |  |  |  |  |  |
| F1 |  | 0.719 |  |  |  |  |  |  |  |  |  |  |
| F5 |  | 0.718 |  |  |  |  |  |  |  |  |  |  |
| F2 |  | 0.707 |  |  |  |  |  |  |  |  |  |  |
| LM3 |  |  | 0.885 |  |  |  |  |  |  |  |  |  |
| LM2 |  |  | 0.870 |  |  |  |  |  |  |  |  |  |
| LM1 |  |  | 0.861 |  |  |  |  |  |  |  |  |  |
| LM4 |  |  | 0.851 |  |  |  |  |  |  |  |  |  |
| LM5 |  |  | 0.777 |  |  |  |  |  |  |  |  |  |
| D1 |  |  |  | 0.779 |  |  |  |  |  |  |  |  |
| D2 |  |  |  | 0.702 |  |  |  |  |  |  |  |  |
| D4 |  |  |  | 0.574 |  |  |  |  |  |  |  | -0.342 |
| D5 |  | -0.300 |  | 0.514 |  |  |  |  |  |  |  | -0.303 |
| D3 |  |  |  | 0.483 |  |  |  |  |  |  |  |  |
| LV5 |  |  |  |  | 0.915 |  |  |  |  |  |  |  |
| LV3 |  |  |  |  | 0.902 |  |  |  |  |  |  |  |
| LV4 |  |  |  |  | 0.883 |  |  |  |  |  |  |  |
| LV2 |  |  |  |  | 0.814 |  |  |  |  |  |  |  |
| LV1 |  |  |  |  | 0.810 |  |  |  |  |  |  |  |
| I2 |  |  |  |  |  | 0.931 |  |  |  |  |  |  |
| I1 |  |  |  |  |  | 0.922 |  |  |  |  |  |  |
| I3 |  |  |  |  |  | 0.889 |  |  |  |  |  |  |
| I4 |  |  |  |  |  | 0.757 |  |  |  |  |  |  |
| I5 |  |  |  |  |  | 0.745 |  |  |  |  |  |  |
| EA1 |  |  |  |  |  |  | 0.896 |  |  |  |  |  |
| EA3 |  |  |  |  |  |  | 0.837 |  |  |  |  |  |
| EA2 |  |  |  |  |  |  | 0.821 |  |  |  |  |  |
| EA5 |  |  |  |  |  |  | 0.718 |  |  | -0.352 |  |  |
| EA4 |  |  |  |  |  |  | 0.681 |  |  | -0.370 |  |  |
| SACN4 |  |  |  |  |  |  |  | -0.818 |  |  |  |  |
| SACN3 |  |  |  |  |  |  |  | -0.807 |  |  |  |  |
| SACN1 |  |  |  |  |  |  |  | -0.763 |  |  |  |  |
| SACN5 |  |  |  |  |  |  |  | -0.738 |  |  |  |  |
| SACN2 |  |  |  |  |  |  |  | -0.614 |  | -0.360 |  |  |
| SACX1 |  |  |  |  |  |  |  |  | -0.874 |  |  |  |
| SACX2 |  |  |  |  |  |  |  |  | -0.839 |  |  |  |
| SACX3 |  |  |  |  |  |  |  |  | -0.679 |  |  |  |
| SACX5 |  |  |  | 0.341 |  |  |  |  | -0.574 |  |  |  |
| SACX4 |  |  |  | 0.369 |  |  |  |  | -0.564 |  |  |  |
| M5 |  |  |  |  |  |  |  |  |  |  | 0.774 |  |
| M2 |  |  |  |  |  |  |  |  |  |  | 0.679 |  |
| M3 |  |  |  |  |  |  |  |  |  |  | 0.652 |  |
| M1 |  |  |  |  |  |  |  |  |  |  | 0.603 |  |
| M4 |  |  |  |  |  |  |  |  |  |  | 0.449 | -0.350 |
| A1 |  |  |  |  |  |  |  |  |  |  |  | -0.887 |
| A2 |  |  |  |  |  |  |  |  |  |  |  | -0.842 |
| A4 |  |  |  |  |  |  |  |  |  |  |  | -0.660 |
| A3 |  |  |  |  |  |  |  |  |  |  |  | -0.591 |
| A5 |  |  |  |  |  |  |  |  |  | -0.324 | 0.345 | -0.449 |

"Extraction Method: Principal Component Analysis. Rotation Method: Oblimin with Kaiser Normalization."

a. Rotation converged in 34 iterations.
