# Supplementary material for: Emotional flexibility and general self-efficacy: A pilot training intervention study with knowledge workers
Source: PLoS One. 2020 Oct 14;15(10):e0237821. doi: 10.1371/journal.pone.0237821 (PMC7556510; doi:10.1371/journal.pone.0237821)
Supplement: Attachment — Submitted filename: 20200728_Response_to_Reviewers_FINAL.docx [file pone.0237821.s007.docx]

Dear Editor,

Thank you very much for offering the opportunity to revise our paper “Emotional Flexibility and General Self-Efficacy. A Pilot Training Intervention Study with Knowledge Workers” for PLOS ONE. Whilst producing a revised draft, we benefitted greatly from the constructive feedback of all reviewers. On the next pages, you find our detailed responses to their feedback.

We also would like to respond here to one of your additional questions: *“Please provide additional details regarding participant consent. In the Methods section, please state why it was not possible to obtain written consent and whether the ethics committee approved this consent procedure described”*

We were acutely aware of the utmost importance to follow careful guidelines in research in general (whether this applies to medical data or not). To abide by these guidelines, we took the following steps:

1.	Medical data collection (not used in the current paper): we made sure to invite a medically schooled professional (MD qualified) to collect this data, following the strict rules of patient data collection that are normal in this profession.

2.	Approval: The overall project and procedures were approved by the internal review board of the university from where the first author carried out this study (Maastricht University in the Netherlands). This paper’s study was performed as the concluding activity to obtain the International Master in Affective Neuroscience of Maastricht University and the University of Florence. Maastricht University was the approval institution.

3.	Participant Consent: The consent issue was emphasized in each step of the study. That is, participants were notified on different occasions about the research elements of the study, and the option to exit, as each and every step in the process is voluntary: (1) at the kick-off call explaining expectations; (2) when completing the survey; (3) during the workshops; and (4) during the HRV measurement when the medical doctor recorded their verbal consent (not used in this study).

We have included the above (without the part referring to the study under #2, in the Methodology section in the revised Manuscript.

We believe we have been able to produce a substantially improved manuscript. We hope you do agree.

Sincerely yours,

Jacqueline Brassey

Arjen van Witteloostuijn

Csaba Huszka

Tobias Silberzahn

Nick van Dam

Response to Editor

Below, we respond to all remarks made by the three reviewers, one by one. We do this in plain font, reproducing the original comment *in italics*, for your convenience.

**Reviewer 1**

*Important note: This review pertains only to ‘statistical aspects’ of the study and so ‘clinical aspects’ [like medical importance, relevance of the study, ‘clinical significance and implication(s)’ of the whole study, etc.] are to be evaluated [should be assessed] separately/independently. Further please note that any ‘statistical review’ is generally done under the assumption that (such) study specific methodological [as well as execution] issues are perfectly taken care of by the investigator(s). This review is not an exception to that and so does not cover clinical aspects {however, seldom comments are made only if those issues are intimately / scientifically related & intermingle with ‘statistical aspects’ of the study}.*

Thank you very much. We highly appreciate your statistical advice, and took notice of that as much as we can. Below, we explain what have done in detail, for each of the points you made.

*Your ABSTRACT is well drafted but assay type. Please note that it is preferable [refer to item 1b of CONSORT checklist 2010: Structured summary of trial design, methods, results, and conclusions] to divide the ABSTRACT with small sections like ‘Objective(s)’, ‘Methods’, ‘Results’, ‘Conclusions’, etc. which is an accepted practice of most good/standard journals [including PLOS]. It will definitely be more informative then, I guess [even if your article type is ‘Research Article’].*

We have followed the guidelines as given to us by PLOS ONE: “Please ensure that your manuscript meets PLOS ONE's style requirements, including those for file naming. The PLOS ONE style templates can be found at:

<https://journals.plos.org/plosone/s/file?id=wjVg/PLOSOne_formatting_sample_main_body.pdf> and <https://journals.plos.org/plosone/s/file?id=ba62/PLOSOne_formatting_sample_title_authors_affiliations.pdf> “

These guidelines do not ask us to separate the Abstract with small sections.

*Overall, this study is almost faultless and the manuscript of this article is excellently drafted.*

Thank you very much. We are pleased to hear you appreciated our article.

*However, I have few suggestions:*

*1. Although use of the ‘tools’ is very appropriate, nature of the data yielded by such tools are generally of ‘ordinal’ level of measurement and so use of non-parametric test(s) is desirable {example Wilcoxon’s Signed Rank test instead of paired ‘t’ test or ‘Spearman’s (rank/non-parametric) correlation coefficient’ instead of ‘Pearson’s correlation coefficient’}.*

Thank you for your feedback. You are indeed right. We have redone the correlations using Spearman’s correlation’s coefficients, and now report Wilcoxon’s Signed Rank rather than paired t tests. The updated tables are included in the revised manuscript. The conclusions remained unchanged.

*2. Note that oftentimes recoding of ‘Likert’ scale responses, {example, lines 324-328: General self-efficacy (GSE) scale includes ten items.& all ten items are rated on a four-point scale: 1 = ‘Not at all true’; 2 = ‘Hardly true’; 3 = ‘Moderately true’; and 4 = ‘Exactly true’} as follows, will be useful. Whenever response options ranged from 1=strongly disagree to 4=strongly agree (or from 1=very bad to 3=neither good nor bad to 5=very good), while using a ‘Likert’ scale responses, recoding [like strongly disagree=(-)2, disagree=(-1), neutral=0, agree=(+)1, strongly agree=(+)2] may yield correct and meaningful ‘arithmetic mean’ which is useful not only for comparison but has absolute meaning, in my opinion. Application of any statistical test(s) assume that meaning of entity used (mean, SD, etc) has a particular meaning. Though ‘α’ [alpha] or most other measures of reliability/correlation will remain same, however.*

Thank you for your feedback. Indeed, centering the responses around zero on Likert and Likert-type scales can facilitate interpretation. Note that we decided to stay in line with the guidelines of the authors of the original scales. Therefore, we used a 6-point scale for the EF and EI scales, as recommended by Rolffs et al. (2018), and the 4-point scale for GSE, as recommended by Schwarzer & Jerusalem (<http://userpage.fu-berlin.de/~health/engscal.htm>).

*3. Note that highly significant (large) value of ‘Pearson’s correlation coefficient’ alone does not imply cause-effect relationship. There are other certain criteria which are to be considered before making any causal inference(s) [particularly refer to line 480 –-- effect is ‘not in a linear way’ always]. Statistical test usually used to assess significance of Pearson’s ‘Correlation coefficient (r)’ is ‘t’ [where t = { r 􀀀 [(n-2) / (1-r2)] }for df=n-2, n is sample size] and here Ho is that the population/standard value of ‘r’ is zero. You need r=0.878 to be significant at 5% if n=5 but you need r=0.273 if n=50 & you need only r=0.088 if n=500. ‘P-value’ heavily depends on sample size. Therefore, it is customary to use the absolute value of ‘r’ guidelines for interpreting positive or negative correlations (and not rely on corresponding ‘P’-value).*

Line 480 in the previous manuscript referred to Table 8 (Improvement vis-à-vis Baseline results). In the revised draft we emphasize even stronger that these are highly exploratory results and that follow-up studies with more power are needed to make any claims as mentioned above. Also, given that this is a pilot study with a small *n*, we are cautious in the whole paper to not overclaim our findings in terms of causality. Clearly, we first need more follow-up studies with more power before we can do that.

*4. Data of table-5, table-8, and table-9 could have been analysed in other better ways [consult a biostatistician for advice].*

In line with the other reviewer’s advice, we opted for downplaying our analyses by making very explicit that what we have here is a (unique) pilot study – nothing more, nothing less. The key strength is the unique setting and design (= an intervention study in the field, with rare access to a real organization and real employees). The key weakness and associated limitation is low power, with an n of only around 30. Hence, after carefully considering your advice, we decided to keep the statistical analyses simple, in the spirit of a low-powered pilot study. Of course, in future work, provided the *n* will be large enough, more sophisticated statistical analyses will be appropriate.

*5. Discussing/highlighting ‘new insights emerged from this study’ [lines 506-28] is a very good practice/idea.*

Thank you very much.

*6. As said in lines 550-52 that “We have not seen any sign of this self-selection in the data”, however, please note you seldom see any sign of self-selection and [should not believe that a similar effect will be achieved in other studies with random assignment] so, this must be examined in future work. I agree with what is said at the end that “Overall, this study contributes by offering insights into the opportunity and feasibility for organizations to respond to the increasing need to invest in their employees’ mental health.”*

Cheers.

**Reviewer 2**

*Reviewer #2: Thank you for submitting this work to PLOS ONE. I found this to be a very interesting experimental work in which the authors have tested whether an intervention to develop emotional flexibility will improve an individual’s emotional flexibility and general self-efficacy. However, there are a couple of key issues that need to be addressed.*

Thank you very much. We are glad you like our work.

*QUESTIONS:*

*Is the manuscript technically sound, and do the data support the conclusions? Has the statistical analysis been performed appropriately and rigorously? The explanation of these two questions can be found in the following comments.*

*MAJOR ISSUES:*

*1. There was substantial attrition in this study, as the authors noted, but the reasons given were not sufficient. While in a workplace setting last minute urgent work may have been a primary reason given by the Author's clients, were there any other characteristics of those individuals who continued compared to those who did not that should/could be mentioned? Attrition was substantial and should be addressed further.*

Thank you for your feedback. We have taken another look at this to see if we could find more information to support our observations. We looked at the characteristics of those showing up for the survey versus participating in the first workshop vs not returning at a later stage.

**For Group 1**: From the group that had signed up, we eventually had 28 completing before-intervention survey1. We had an attrition of six participants (21%) who did not come to the first workshop (*n*=22 for survey1 and workshop1 participants) and lost another five (18%) for the after-intervention survey2 (total *n*=17 for both surveys and at least one workshop, total attrition vis-à-vis first survey: 39%). The six who did not show up but who did fill out the survey scored lower on mean EF (3.02) and higher on mean IF (3.25), and slightly lower on mean GSE (2.95) [same results for the ‘remainers’: EF (3.84), IF (2.685) and GSE (3.18)]. Hence, we can reasonably argue that the six who could not come would actually need the workshop more, on average, than the employees who came. We did get cancellations via e-mail for some of those six and know this had to do with personal circumstances or work challenges. One explanation we can think of is that having lower flexibility, higher inflexibility, and lower GSE can perhaps lead to less courage to push back when work challenges arise, and not daring to withstand leadership. But we really have only six datapoints for this without any further information. Hence, all this is highly speculative. In terms of demographics: The six who did not show up consisted of three females and three males. One of these participants was from the age group 18-24, four of these participants were from the age group 25-34 years, and one from 34-44 years. There were five participants with the German nationality and one with the Austrian nationality. Finally, one participant indicated to have a Bachelor’s degree, three a Master’s degree and two had a PhD degree. This is not substantially different from their colleagues who did show up.

**For Group 2**: From the group who had completed survey1 (*n*=31), we eventually had 27 filling in the second survey (representing the control group). Subsequently, 23 (15% attrition vis-à-vis the second survey) of those participants came to the first workshop, and we lost another two for the after-intervention survey2 (total *n*=21 for both surveys and at least one workshop; total attrition of 22% vis-à-vis second survey). From the eight participants who filled out the survey at the start but who eventually did not join the first workshop, we looked at the averages of that group at the start compared to the rest of the group. The comparisons were as follows: EF (‘joiners’: 3.32; attrition: 4.16), EI (‘joiners’: 2.95; attrition: 2.95), and GSE (‘joiners’: 3.25; attrition: 3.23). The ‘joiners’ started with a lower EF than their colleagues who did not come, eventually, but the EI and GSE scores were the same. This may indicate that they realized, perhaps, that they were not really interested after all. As for Group 1, from some we got cancellations indicating they had to cancel, with reasons as above. The attrition group demographics were as follows: three of these were females, and five males; six were in the age group 25-34 years, and two in the age group 34-44. All were of German nationality. Seven of them had a Master’s degree and one a PhD degree. Again, the differences with the ‘joiners’ were not large.

3. As an aside, we would like to ‘nuance’ our statement in the original draft that there was huge attrition. The attrition was not so much related to survey response itself; these were actually not that bad at all, as mentioned in our previous response. For Group 1, total attrition was 39% vis-à-vis first survey and including all who joined at least one workshop; for Group 2, this was 22%. The biggest attrition was seen regarding joining workshops, and not so much for completing surveys. Four of the eight who did not join workshops still filled out the last survey, an indication that they were committed to actually participate. Key here is that surveys can be done everywhere anytime, since they were online. Given their very busy jobs and the agreement that the job came first, we should not be surprised that quite a few could not physically join the workshop after all.

Notwithstanding the points made above, we have to be careful to generalize any of our findings beyond our pilot. Indeed, as you rightly point out, given the low *n* in combination with attrition, we clearly have a pilot study here (see below). For sure, more work is needed in the future. We have added a summary of the above in the revised manuscript.

*2. The substantial attrition has led to additional issues pertaining to the statistical analyses and the conclusions derived by the authors. The most concerning issue is sample size. The statistical analyses used with such a small sample size were not sufficient, or appropriate, and do not support the conclusions derived by the authors. The conclusions are overstated, and this data would be best framed as pilot study data. Running correlations with such a small sample size is problematic. For example, these results could be driven by one or two outliers rather than being a legitimate effect. Additionally, running PCA with such a small sample size (despite the returned results) is potentially inappropriate. The authors have broken down the baseline EF into buckets some of which had just 1 participant - this is not sufficient to be making conclusions relating to improvements from baseline etc. Finally, we should reconsider using these types of statistical analyses for such small sample sizes and use more appropriate analyses with which to form conclusions.*

*The primary issues with this work suggest that it would benefit from being re-framed as a pilot study, or a technical report in which the authors are providing some evidence for this methodology to be used in a larger scale study. The introduction and discussion read like a technical report with very little background research but more prominently discussing the methodology and the foundations of the methodology. I would advise the authors to either collect more data, or to re-frame this paper as a pilot study, or technical document.*

Thank you for your feedback. We fully agree and have renamed this study as a ‘pilot study’.

**Reviewer 3**

*Reviewer #3: I’ve read with interest and care your paper titled “Emotional Flexibility and General Self-Efficacy: A training Intervention Study with Knowledge Workers”. I commend your choice to focus on emotional flexibility. I do believe that the development of emotional flexibility among knowledge workers is crucial for their well-being, especially nowadays and in the future*

Thank you very much. We appreciate this and are glad you liked our work.

*In this review, I will describe the few concerns I have with the current manuscript and try and provide some observations that may assist you in strengthening the study and in pursuing this interesting line of research.*

*1. Please clarify what was the time frame you have used when collecting responses for the 12 dimensions of the MPFI. Was it “on general” or “in the last two weeks” or something else?*

In line with the recommendations by Rolffs et al. (2018), we used the following for these MPFI items: “When answering the following questions, please reflect on the past two weeks at work.”

*2. Please explain in more detail the possible reasons why the scores on ‘Purpose and values’, and ‘No purpose and values have changed for the control group*

We describe that in Lines 280-281: *“During this period, the second group was not going through workshops, but was exposed to an informal campaign around better performance at work”.* This campaign included emphasis on ‘purpose’ and personal well-being. We were wondering whether this group was more impacted by this, and therefore had improved on this dimension because of this emphasis. But if that would have been the case, this should also apply to Group 1, who was also exposed to this campaign (as all employees were). We also see an improvement in this period for Group 1, but we cannot say if that was as a result of the campaign (to which also Group 1 was exposed) or the treatment. So, we then looked at the baseline average for this dimension for Group 1 (*n*=21) compared to the control group (*n*=27). We found that the baseline average for Group 1 on this dimension for “Values” was higher than the baseline average for the Control Group (G1: 4.01 vs CG: 3.19), observing the same pattern for “Lack of contact with Values” for which Group 1 started at a lower (hence less inflexible) level (G1: 2.94 vs CG: 3.62). Both groups improved during period 1, for ‘V’ (G1: 4.19 vs CG: 3.65) and for ‘LV’ (G1:2.60 vs CG: 3.622), but the change for G1 was not significant. The Control Group, which turned into Group 2 (G2) later, but with a smaller *n*-size (*n*=17), subsequently improved on the ‘Values’ dimension when going through the treatment (“Values” from 3.39 to 3.78 and “Lack of Contact with Values” from 3.32 to 2.98). Also, the ‘Lack of Contact with Values’ dimension improved when going through the treatment, although improvement is not significant. Hence, even though there was a change without treatment, we saw again a change during treatment.

Looking at the 17 participants consistently (see the table below), we observe that Group 2 went through a change like follows: improving consistently over time, hence AND during the period where there was no treatment, but also AGAIN after the treatment.

So we cannot be sure why the change happened in the pre-treatment period, but we can see that there was also still an effect after the treatment for Group 2. We now explain all this in the revised draft.

*In sum, this is an interesting paper. I wish you good luck as you continue working in this area and hope that my few comments are taken in the positive and constructive manner in which they are intended.*

Thank you very much for your feedback. Much appreciated.
